# Supplementary material for: Empirical methods for controlling false positives and estimating confidence in ChIP-Seq peaks
Source: BMC Bioinformatics. 2008 Dec 5;9:523. doi: 10.1186/1471-2105-9-523 (PMC2628906; doi:10.1186/1471-2105-9-523)
Supplement: Additional File 1 — A variety of html documents from the USeq web site detailing the available applications, their best usage, output file type descriptions, command line menus, etc. [file 1471-2105-9-523-S1.zip › USeqUserGuides/JavaDoc.zip!/JavaDoc/index-files/index-8.html]

H-Index


|  |  |  |
| --- | --- | --- |
| **Overview** \| Package \| Class \| Use \| **Tree** \| **Deprecated** \| **Index** \| **Help** | |  |
| **PREV LETTER**   **NEXT LETTER** | **FRAMES**    **NO FRAMES**     **All Classes** |  |


A B C D E F G H I J L M N O P Q R S T U V W X Z 

---


## **H**

**hashMiRNATable(File)** - Static method in class expr.ParseMiRNATable: **hashSetToFloat(HashSet<Float>)** - Static method in class util.gen.Num: Given a HashSet of Floats returns an array of float. **hashSetToInt(HashSet<Integer>)** - Static method in class util.gen.Num: Given a HashSet of Integers returns an array of int. **hashSetToStringArray(HashSet)** - Static method in class util.gen.Misc: Converts a hash to a String[] by direct cast. **header** - Static variable in class util.bio.wrappers.Primer3Pick: **HeatMapMaker** - Class in edu.utah.seq.data: Converts a SmoothingWindow[] into a heat map specific bar file for import into IGB. **HeatMapMaker(int, float)** - Constructor for class edu.utah.seq.data.HeatMapMaker: **HeatMapMakerPosNeg** - Class in edu.utah.seq.data: Converts a SmoothingWindow[] into a heat map specific bar file for import into IGB. **HeatMapMakerPosNeg(int, float, float)** - Constructor for class edu.utah.seq.data.HeatMapMakerPosNeg: **hexamerGenerator()** - Static method in class util.bio.xamer.XamerCounter: **HierarchicalClustering** - Class in util.bio.cluster: Calculates a Pearson correlation coefficient between all pairs of serialized float[]s, uses hierarchical clustering to group like arrays. **HierarchicalClustering(String[])** - Constructor for class util.bio.cluster.HierarchicalClustering: For stand alone use. **HierarchicalClustering(double, int, StringBuffer, File, boolean)** - Constructor for class util.bio.cluster.HierarchicalClustering: For integration with CelFileQualityControl App. **HierarchicalClustering(File[])** - Constructor for class util.bio.cluster.HierarchicalClustering: For integration with Correlate App. **HierarchicalClustering(File[], File)** - Constructor for class util.bio.cluster.HierarchicalClustering: For integration with CelProcessor. **highestScoringWindow(int, int)** - Method in class edu.utah.seq.data.HeatMapMaker: Looks forward at overlapping windows, returns highest score for the given bp position. **highestScoringWindow(int, int)** - Method in class igb.util.Windows2HeatMapSgr: Looks forward at overlapping windows, returns highest score for the given bp position. **highestScoringWindow(int, int)** - Method in class trans.main.WindowBlockMaker: Looks forward at overlapping windows, returns highest score for the given bp position. **Histogram** - Class in util.gen: Generates histograms. **Histogram(double, double, int)** - Constructor for class util.gen.Histogram: **Histogram(double[], int, double)** - Constructor for class util.gen.Histogram: Given an array of scores, creates a histogram of set number of bins, trims tails by a set fraction of the data. **HistogramSgrOrGrFile** - Class in igb.util: **HistogramSgrOrGrFile()** - Constructor for class igb.util.HistogramSgrOrGrFile: **HouseBean** - Class in util.apps: **HouseBean(String[])** - Constructor for class util.apps.HouseBean: **HTML** - Class in util.gen: Static methods for HTML related tasks. **HTML()** - Constructor for class util.gen.HTML: **HTMLPath** - Static variable in class bioroot.Util

---


|  |  |  |
| --- | --- | --- |
| **Overview** \| Package \| Class \| Use \| **Tree** \| **Deprecated** \| **Index** \| **Help** | |  |
| **PREV LETTER**   **NEXT LETTER** | **FRAMES**    **NO FRAMES**     **All Classes** |  |


A B C D E F G H I J L M N O P Q R S T U V W X Z 

---
